# Global urbanization and the neglected tropical diseases

**DOI:** 10.1371/journal.pntd.0005308

**Published:** 2017-02-23

**Authors:** Peter J. Hotez

**Affiliations:** 1 Sabin Vaccine Institute and Texas Children’s Hospital Center for Vaccine Development, Departments of Pediatrics and Molecular Virology and Microbiology, National School of Tropical Medicine, Baylor College of Medicine, Houston, Texas, United States of America; 2 James A Baker III Institute for Public Policy, Rice University, Houston, Texas, United States of America; 3 Department of Biology, Baylor University, Waco, Texas, United States of America; 4 Scowcroft Institute of International Affairs, Bush School of Government and Public Service, Texas A&M University, College Station, Texas, United States of America; Swiss Tropical and Public Health Institute, SWITZERLAND

## Increasing urbanization in both developing and developed countries could promote the emergence of a new set of neglected tropical diseases (NTDs)

**Fig 1:**
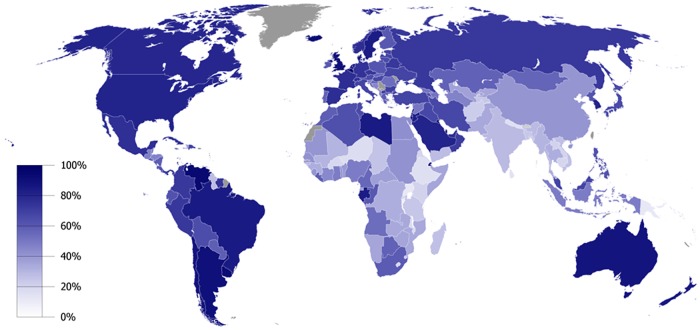
Global urbanization map showing the percentage of urbanization by country in 2006. Public domain image available here: https://commons.wikimedia.org/wiki/File:Urbanized_population_2006.png.

In 2014, the United Nations (UN) launched an important report on global trends in urbanization [1]. The study found that beginning in 2007, for the first time in human history, more people live in urban than rural areas, with estimates that by 2050 approximately two-thirds of the world’s population will be urbanized [1, 2]. As shown in Fig 1, the Western Hemisphere (especially North America) exhibits the highest percentage of urban dwellers, in addition to Australia and selected other areas, but the UN report also predicts some important trends in Africa and Asia.

For example, it was found that much of the future increase in urban populations will happen in Asia and Africa, where by the year 2050, 64% (up from 48% in 2014) and 56% (up from 40% in 2014) will be urbanized, respectively [1]. China, India, and Nigeria will comprise almost 40% of the future expansion in urban populations from now until 2050 [1]. During this period, new “megacities”—cities with populations that exceed 10 million people—will be formed. Most of the new megacities will be found in Asia, Africa, and Latin America where NTDs are highly endemic [1], including Mumbai, Delhi, and Kolkata (India), Dhaka (Bangladesh), Kinshasa (Democratic Republic of Congo), Lagos (Nigeria), Luanda (Angola), Dar es Salaam (Tanzania), Bogota (Colombia), and Lima (Peru) [1]. By 2030, the UN estimates that there will be 41 such megacities globally [1].

We can predict that urbanization will produce both positive and negative effects on the urban populations in the Global South. On the positive side, urbanization is often linked to culture, commerce and economic productivity, greater life expectancy, higher levels of education and literacy, increased access to social services, better access to health care, and an overall higher quality of life [1]. At the same time, rapid urbanization can also fail to sustain healthy populations when it outstrips clean water reserve and sewage management systems or when urban poverty produces unhealthy diets and diminished physical activity, low-quality housing, and environmental degradation, together with exposure to air, noise, and other forms of pollution [1– 3]. Crowding can also be an important factor. Through such mechanisms, the effects can be devastating in terms of disease transmission and even thwart Sustainable Development Goals (SDGs).

In recognition of these factors, in 2010, WHO chose urbanization as its theme for World Health Day [2]. Rapid global urbanization over the next few decades has potentially important implications for the rise of NTDs, as well as NTD and noncommunicable disease (NCD) comorbidities. While an important feature of NTDs is their disproportionate impact on populations living in rural poverty, there are selected diseases that primarily affect the poor in urban settings (Box 1) [4]. Described below are some of the major NTDs emerging in urban environments over the last three years.

**Box 1. High prevalence and incidence NTDs emerging in urban environments**

- Arbovirus infections transmitted by *Aedes aegypti*
  - Dengue
  - Chikungunya
  - Zika virus infection
- Canine rabies
- Leptospirosis, cholera, and typhoid fever
- Schistosomiasis and soil-transmitted helminthiases
- Chagas disease and leishmaniasis
- Intestinal protozoan infections

### Neglected virus infections: Arboviruses and rabies

*Aedes aegypti* is an urban-dwelling mosquito, specifically adapted to humans and responsible for the transmission of dengue, chikungunya, yellow fever, and Zika virus infection. The dramatic emergence of Zika virus infection in Brazil in 2015, struck the crowded and impoverished northeastern city of Recife particularly hard [5], and there are concerns about Zika now traveling to additional New World and even Old World cities [6]. Since the end of 2013, chikungunya is also now affecting some of those same cities in the Americas, while, according to the Global Burden of Disease Study 2015 (GBD 2015), the number of global dengue cases has increased from approximately 33 million to 80 million incident cases annually over the last decade [7]. Similarly, urban rabies transmitted from dogs remains an important cause of mortality, although there has been almost a 50% decline over the last decade, with 17,400 deaths in 2015 according to the GBD 2015 [8]. While there are no global data comparing urban versus rural incidence rates of canine-transmitted rabies, in Delhi, India, urban slums were recently shown to exhibit higher dog bite incidence rates compared with rural slums, with the majority of both populations not receiving rabies postexposure prophylaxis [9].

### Neglected bacterial infections: Leptospirosis, cholera, and typhoid fever

Leptospirosis has emerged as an important urban bacterial zoonosis from rat and dog urine, especially in the favelas of Brazil’s cities, such as Salvador where it is an important yet underreported cause of acute febrile illness [10]. The spatio-temporal determinants of infection there have been studied extensively [11]. Urban leptospirosis has also been reported in Nairobi [12]. Although not currently incorporated into the GBD 2015, a recent effort to determine the global burden of leptospirosis estimates approximately 1 million annual cases resulting in almost 3 million disability-adjusted life years [13]. However, the study did not report the percentage of cases found in urban versus rural environments. Similarly, there are multiple reports of cholera, typhoid fever, and other enteric infection outbreaks in urban slums and in the settings of poor urban planning or following urban natural disasters [14–18]; however, there are no published global burden data that differentiate the urban and rural outbreaks.

### Neglected parasitic infections

Urban schistosomiasis and ascariasis (as well as other soil-transmitted helminthiases) have been reported from Africa [19–21] and Latin America [22, 23]. Interestingly, the Global Atlas of Helminth Infection found that ascariasis and trichuriasis transmission is highest in peri-urban rather than either urban or rural settings [24]. Some urban communities can also sustain lymphatic filariasis (LF) transmission [25], although it was shown recently that rural to urban migrations due to the conflicts in Sierra Leone and Liberia could not sustain LF transmission [26]. An outbreak of urban Chagas disease has also been reported from Venezuela and Peru [27, 28]. Urban zoonotic visceral leishmaniasis from dogs has been reported from Argentina and elsewhere in the Americas, with low-quality housing, crowding, and dog ownership representing some of the key risk factors [29, 30]. In India and elsewhere, vivax malaria also represents an important neglected parasitic infection, which has been refractory to control measures [31]. Giardiasis and other enteric protozoan infections have also been shown to cluster in urban environments [21, 32].

### NTD and NCD comorbidities

Yet another phenomenon we might expect to see with increasing frequency in the new urban megacities is the increasing overlap of NTDs with NCDs. For example, in India a new high mortality has been seen in dengue patients with underlying hypertension and diabetes [33]. As NCDs expand in poor countries due to tobacco and lifestyle changes, we can expect to see further examples of such NTD comorbidities.

Overall, there is a dearth of information about the urban transmission of NTDs and very few disease burden estimates that distinguish urban versus rural modes of transmission. As global urbanization continues to increase, there is going to be an urgent need for such studies. By 2050, with most of the global population living in cities, we will need to better understand how NTDs and other poverty-related neglected diseases flourish in urban environments.

The UN is beginning to shape new public policies for global urbanization, which include programs for balanced urban growth and spatial distribution, sustainability, and timely collection of data required for urban planning [1]. The findings of significant and serious NTDs in urban areas mean that these diseases will also need to be considered as urban areas and megacities strive to meet their SDGs. Arbovirus infections, leptospirosis, cholera, and typhoid fever, vector-borne parasitic infections such as schistosomiasis, Chagas disease, leishmaniasis, and vivax malaria, and NTD–NCD comorbidities each represent the product of urban planning breakdowns and unchecked growth. Without adequate public health measures and research and development for new drugs, diagnostics, and vaccines, we can expect that these diseases will continue to thwart sustainable urban growth in the coming decades.
